# The landscape of RNA-chromatin interaction reveals small non-coding RNAs as essential mediators of leukemia maintenance

**DOI:** 10.1038/s41375-024-02322-7

**Published:** 2024-06-28

**Authors:** Haiyang Yun, Julian Zoller, Fengbiao Zhou, Christian Rohde, Yi Liu, Maximilian Felix Blank, Stefanie Göllner, Carsten Müller-Tidow

**Affiliations:** 1 Department of Medicine V, Hematology, Oncology and Rheumatology, University Hospital Heidelberg, Heidelberg, Germany; 2 The Robert Bosch Center for Tumor Diseases, Stuttgart, Germany; 3 Molecular Medicine Partnership Unit, European Molecule Biology Laboratory (EMBL), Heidelberg, Germany; 4 Division Proteomics of Stem Cells and Cancer, German Cancer Research Center (DKFZ), Heidelberg, Germany; 5 National Center for Tumor Diseases (NCT), Heidelberg, Germany

**Keywords:** Acute myeloid leukaemia, Cancer epigenetics

## Abstract

RNA constitutes a large fraction of chromatin. Spatial distribution and functional relevance of most of RNA-chromatin interactions remain unknown. We established a landscape analysis of RNA-chromatin interactions in human acute myeloid leukemia (AML). In total more than 50 million interactions were captured in an AML cell line. Protein-coding mRNAs and long non-coding RNAs exhibited a substantial number of interactions with chromatin in *cis* suggesting transcriptional activity. In contrast, small nucleolar RNAs (snoRNAs) and small nuclear RNAs (snRNAs) associated with chromatin predominantly in *trans* suggesting chromatin specific functions. Of note, snoRNA-chromatin interaction was associated with chromatin modifications and occurred independently of the classical snoRNA-RNP complex. Two C/D box snoRNAs, namely *SNORD118* and *SNORD3A*, displayed high frequency of *trans*-association with chromatin. The transcription of *SNORD118* and *SNORD3A* was increased upon leukemia transformation and enriched in leukemia stem cells, but decreased during myeloid differentiation. Suppression of *SNORD118* and *SNORD3A* impaired leukemia cell proliferation and colony forming capacity in AML cell lines and primary patient samples. Notably, this effect was leukemia specific with less impact on healthy CD34+ hematopoietic stem and progenitor cells. These findings highlight the functional importance of chromatin-associated RNAs overall and in particular of *SNORD118* and *SNORD3A* in maintaining leukemia propagation.

## Introduction

The mammalian genome encodes a complex repertoire of transcripts, comprising a substantial number of regulatory RNAs that play crucial roles in governing the transcriptome, epigenome, and epitranscriptome [1, 2]. The regulatory RNA pool is primarily composed of non-coding RNAs (ncRNAs), including long ncRNAs (lncRNAs) that exceed 200 nucleotides, and small ncRNAs (less than 200 nucleotides) such as microRNAs (miRNAs), Piwi-interacting RNAs (piRNAs), small nuclear RNAs (snRNAs), and small nucleolar RNAs (snoRNAs) [3]. Two types of ncRNA, namely lncRNA and miRNA, have been extensively characterized for their pivotal roles in modulating gene expression, through transcriptional or posttranscriptional regulatory mechanisms [4, 5]. The transcriptional regulation mediated by lncRNAs involves association with histones, chromatin modifiers, transcription factors, or mRNA transcripts, thereby modulating the activity and/or stability of these molecules [5, 6]. Recent technological advancements have uncovered additional functions of ncRNAs in mediating or maintaining the three-dimensional structure of genome, implementing another layer to their multifaceted regulatory properties [7–9]. Extensive studies have explored the role of RNA molecules in modulating chromatin in normal tissue development as well as disease pathology, especially in cancer [10, 11]. However, a comprehensive understanding of regulatory RNAs, particularly ncRNAs, in chromatin modulation in *trans*- or *cis*-acting manner in the context of cancer is yet to be established.

Acute myeloid leukemia (AML) is a hematological malignancy characterized by impaired hematopoiesis that affects the myeloid lineage [12]. Its clinical presentation is miscellaneous due to heterogeneous molecular drivers responsible for the disease initiation and progression, posing a challenge for the cure of AML [13]. While a compendium of chromatin regulators driving aberrant transcription in AML have been uncovered [14, 15], our understanding of RNA molecules associated with chromatin and possessing regulatory potential in this context is limited. Nonetheless, the investigation of chromatin regulatory RNAs in AML is of great interest. For instance, the lncRNA *HOTTIP* associating with the *HOXA* locus promotes myeloid leukemogenesis by modulating chromatin architecture [16].

In this study, we conducted a comprehensive analysis of the RNA-chromatin interactome in AML cells to identify chromatin-associated ncRNAs with regulatory functions in AML. We then analyzed chromatin association patterns for different RNA classes and their relationship with chromatin modifications. These analyses allowed us to identify candidate chromatin- associated ncRNAs differentially expressed in normal hematopoietic differentiation as well as in leukemia transformation. In addition, loss-of-function assays revealed the essential role of these regulatory ncRNAs on leukemia cell propagation. Overall, our findings expand our understanding of the role of regulatory RNAs in AML biology and offer potential insights for the discovery of novel therapeutic targets in AML treatment.

## Results

### Characterizing the RNA-chromatin interactome in leukemia cells

**Fig. 1:**
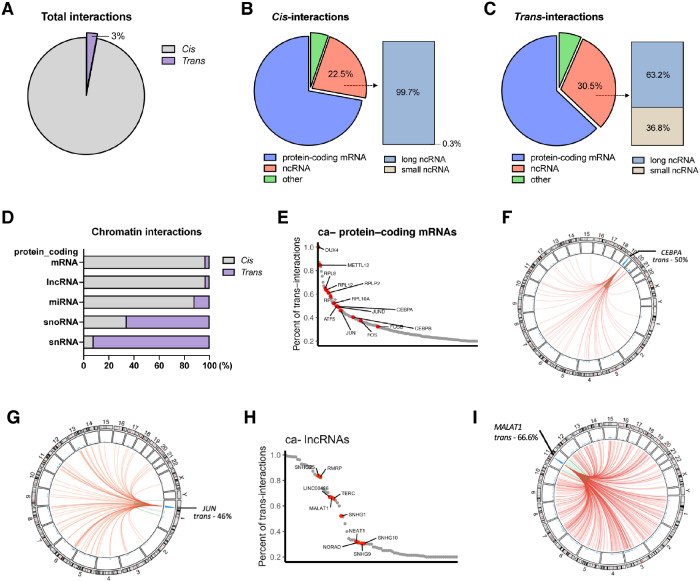
Charting the RNA-chromatin interactome in MV4-11 cells. **A** Proportion of *cis*- or *trans*-interactions detected by iMARGI in MV4-11 cells. **B** Proportion of chromatin interactions associated with diverse RNA classes among *cis*-interactions and **C** *trans*- interactions. **D** Percentage of *cis*- or *trans*-interactions for each coding or non-coding RNA class. **E** *Trans*-acting protein-coding mRNAs. Representative genes are in red and labeled. **F** Circos plot showing genome-wide interactions between *CEBPA* mRNA or **G** *JUN* mRNA and chromatin. Red arcs, *trans*-interactions; green arcs, *cis*-interactions; blue track, interaction frequencies. **H** *Trans*-acting lncRNAs, illustrated as in **E**. **I** Circos plot showing genome-wide interactions between *MALAT1* mRNA and chromatin, illustrated as in **F**.

We employed the in situ mapping of RNA-genome interactome (iMARGI) methodology [17, 18] for genome-scale profiling of chromatin-associated RNAs (caRNAs) in MV4-11 cells, a representative human AML cell line carrying *Flt3-ITD* and *MLL-AF4*, among the most common oncogenes in adult and pediatric leukemias, respectively. iMARGI performs crosslinking between spatially proximal RNA and DNA, followed by in situ ligation with a bridge linker to form RNA-linker-DNA chimeras. These hybrid sequences are further converted into DNA sequences for NGS library preparation, enabling the capture of RNA-DNA interaction pairs by sequencing both ends. This approach allowed for the comprehensive detection of RNAs interacting either with the chromosome of their origin (intra-chromosomal, in *cis*) or with other chromosomes (inter-chromosomal, in *trans*). We generated and sequenced a total of four iMARGI libraries, yielding over 1 billion (1.15 ×10^9^) paired-end reads, which resulted in the capture of approximately 52.1 million valid RNA-chromatin interactions. At a global scale, *cis*-interactions were prevalent with 97%, whereas about 3% (approximately 1.6 million interactions) occurred in *trans* (Fig. 1A). Overall, protein-coding mRNAs dominated the interactome with 37.4 million detected interactions. With a lower frequency, ncRNAs accounted for 22.5% and 30.5% of all *cis*- and *trans*-interactions, respectively (Fig. 1B, C). Notably, lncRNA constituted nearly all (99.7%) of the ncRNA-chromatin *cis*-interactions. In stark contrast, only near two-thirds (63.2%) of ncRNA-chromatin *trans*-interactions were classified as lncRNAs, whereas 36.8% fell into the category of small ncRNAs (Fig. 1B, C). Protein-coding mRNAs, lncRNAs, as well as miRNAs demonstrated a preference for *cis*-interactions (Fig. 1D). Conversely, two classes of small ncRNA, namely small nuclear RNA (snRNA) and small nucleolar RNA (snoRNA), primarily interacted with chromatin in *trans* (Fig. 1D and Supplementary Table 1). These findings suggested that the vast majority of mRNAs and lncRNAs is found at or close to the respective sites of transcription. However, snoRNAs and snRNAs are spread throughout the chromatin which hinted at specific functions in chromatin.

We identified a subset of 207 protein-coding mRNAs (1%) and 91 lncRNAs (0.5%) with *trans*- acting features. These RNAs were characterized by frequent chromatin association ( ≥ 10 interactions) and a substantial proportion of *trans*-interactions ( ≥ 20% of all interactions) (Supplementary Fig. 1A, B). Notably, the *trans*-acting protein-coding mRNAs were enriched for transcription factors (e.g., *DUX*, *CEBPA*/*CEBPB*, *JUN* and *FOS*) and ribosomal protein genes (e.g., *RPL8*, *RPL12* and *RPLP2*) (Fig. 1E–G and Supplementary Fig. 1C). Among the *trans*-acting lncRNAs detected, we observed the presence of *MALAT1* (Fig. 1H, I), a cancer-related lncRNA previously implicated in metastasis, chromatin association and modulation [19, 20]. The *trans*-interactions between protein-coding mRNAs or lincRNAs and chromatin were further verified by quantitative PCR of the iMARIGI library (Supplementary Fig. 1D–F). Additionally, several of these *trans*-acting protein-coding mRNAs and lncRNAs were also found in HEK293T cells [18] (Supplementary Fig. 1G–I).

### Small ncRNAs predominantly interact with chromatin in *trans*

**Fig. 2:**
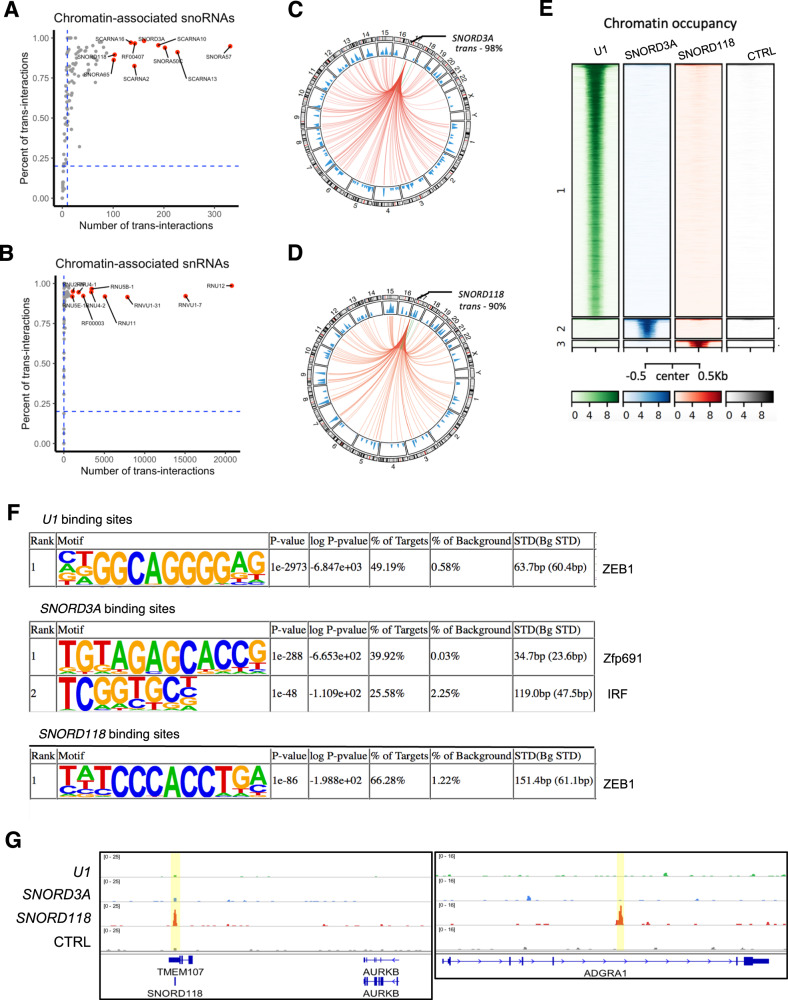
Small ncRNAs predominantly interact with chromatin in *trans.* **A** *Trans*-acting snoRNAs and **B** snRNAs. Top 10 genes with most enriched *trans*-interactions are highlighted in red and labeled. Cut-off is set for chromatin interaction ≥10 and proportion of *trans*-interactions ≥20% of all interactions. **C** Circos plot showing genome-wide interactions between *SNORD3A* RNA or **D** *SNORD118* RNA and chromatin. **E** Chromatin occupancy enrichment of *U1*, *SNORD3A*, and *SNORD118* revealed by ChIRP. **F** De novo motif analysis for *U1*, *SNORD3A*, and *SNORD118* interaction sites. **G** Exemplar loci with *SNORD118* chromatin occupancy. Left, proximal region; Right, distal region.

Further analysis identified a high prevalence of chromatin-associated snRNAs and snoRNAs localized in *trans* (Fig. 2A, B, Supplementary Fig. 2A, B and Supplementary Table 2). The abundance of snRNAs engaged in *trans*-interactions provides support for their crucial role in guiding pre-mRNA splicing [21]. The top-ranked snRNAs exhibiting high number of *trans*-interactions with chromatin included constituents of both the minor spliceosome, namely *RNU12* (*U12*) and *RNU11* (*U11*), as well as of major spliceosome, including *RF00003* (*U1*), *RNVU1* (*U1* variants), *RNU2* (*U2*), *RNU4* (*U4*) and *RNU5* (*U5*) (Fig. 2A). Similarly, the identification of the top enriched *trans*-acting snoRNAs reported two C/D box snoRNAs (*SNORD3A*, *SNORD118*), four H/ACA box snoRNAs (*SNORA57*, *SNORA50C*, *RF00407/SNORA50* and *SNORA65*), and four small Cajal body-specific RNAs (scaRNAs) (*SCARNA13*, *SCARNA10*, *SCARNA2* and *SCARNA16*) which contain both C/D and H/ACA box elements (Fig. 2B–D). Notably, *SNORD3A* and *SNORD118* represent non-canonical C/D box snoRNAs that lack the function of guiding 2-O-methylation of ribosomal RNAs. The chromatin association of *U1*, *SNORD3A*, and *SNORD118* was confirmed by Chromatin Isolation by RNA purification (ChIRP), a method that utilizes capture probes to pull down the target RNA molecules and their associated DNA elements [19, 22]. All three small ncRNAs exhibited specific chromatin occupancy patterns (Fig. 2E and Supplementary Table 3). The binding of snoRNAs was observed to be associated with transcription factor binding motifs such as ZEB1 (for *U1* and *SNORD118*) and Zfp691 (for *SNORD3A*) (Fig. 2F). The specific binding of *U1*, *SNORD3A*, or *SNORD118* at both proximal loci of their origin and distal regions was verified at exemplary loci (Fig. 2G and Supplementary Fig. 2C,D).

### snoRNA-chromatin interaction involved chromatin modification in a snoRNP-independent manner

**Fig. 3:**
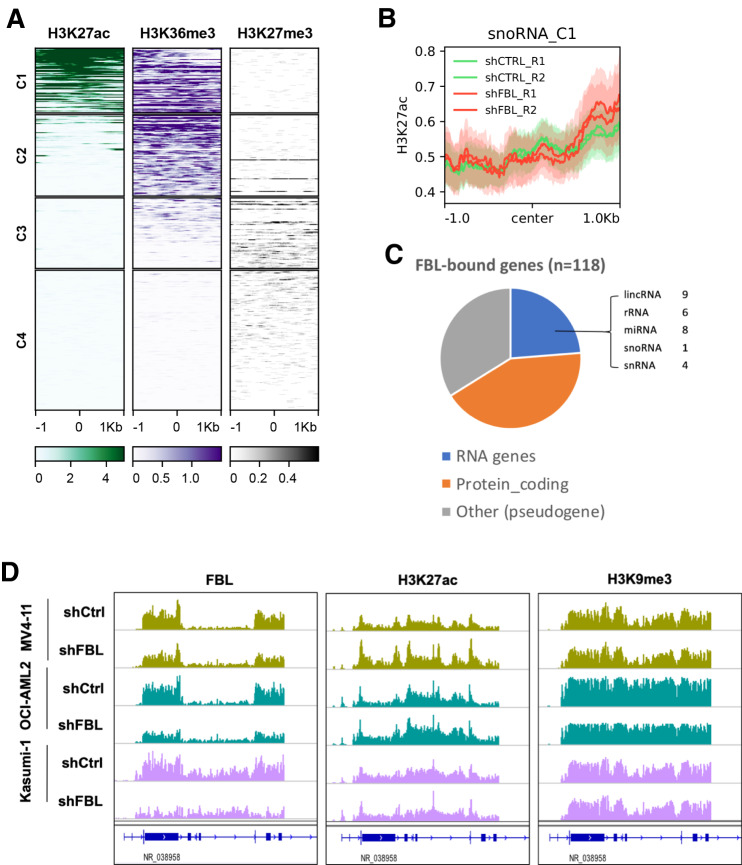
snoRNA-chromatin interaction involved chromatin modifications. **A** Clustering of snoRNA binding sites by enrichment of diverse histone modifications. **B** H3K27ac signals at snoRNA interaction C1 region in MV4-11 cells with *FBL* knockdown versus control. **C** Fibrillarin-bound genes revealed by ChIP-seq in MV4-11 cells. **D** Enrichment of Fibrillarin occupancy, H3K27ac, and H3K9me3 at exemplar loci (a rDNA region) in multiple AML cell lines with or without *FBL* silencing.

The prevalence of snRNA- and snoRNA-chromatin interaction in *trans* suggests modulatory roles of snRNA and snoRNA on chromatin. Therefore, we next determined whether snRNA- and snoRNA-chromatin interactions were associated with specific chromatin modification states. We overlayed RNA-interacting genomic regions determined by iMARGI with chromatin modification status identified by ChIP-Seq with specific antibodies (H3K4me3, H3K27ac, H3K9ac, H3K79me3, H3K36me3, and H3K27me3, RNA Pol II and BRD4). Both snRNAs and snoRNAs displayed a fraction of binding sites which were enriched for active chromatin states as characterized by H3K4me3, H3K27ac and H3K9ac. These sites were enriched for low or high signals of BRD4 and RNA Pol II occupancy at snRNAs or snoRNAs interaction sites, respectively (Supplementary Fig. 3A, B). Further focused analysis on key chromatin marks categorized all regions of snoRNA interactions into four groups (C1-4) (Fig. 3A). Among them, the C1 regions (Supplementary Table 4) showed high levels of H3K27ac and H3K36me3, indicating active transcription at these sites. Given that snoRNAs typically associate with a group of proteins to form small nucleolar ribonucleoprotein (snoRNP) complex, we investigated the relevance of snoRNP in snoRNA-chromatin interactions. To do so, we generated MV4-11 cells with inducible knockdown of Fibrillarin (Supplementary Fig. 3C), encoded by the *FBL* gene, a key component of C/D box snoRNP with enzymatic activity responsible for catalyzing the 2′-O-methylation of ribosomal RNAs. Importantly, loss of *FBL* did not affect H3K27ac signals at snoRNA-interacting C1 regions (Fig. 3B), which suggests Fibrillarin was dispensable for maintaining active chromatin at snoRNA-chromatin contact sites. Further, we performed chromatin immunoprecipitation coupled with high throughput sequencing (ChIP-seq) on Fibrillarin, to profile all Fibrillarin-bound sites across the genome in MV4-11 cells. Fibrillarin occupancy was observed primarily proximal to 118 gene loci, with approximately one quarter located near ribosomal RNA (rRNA) genes and one third close to protein-coding genes (Fig. 3C and Supplementary Table 5). Though knockdown of *FBL* depleted Fibrillarin binding at rDNA regions, active chromatin states marked by H3K27ac remained unchanged (Fig. 3D). Similar results were obtained in two other AML cell lines, Kasumi-1 and OCI-AML2, implying that classical FBL-involved snoRNP complex does not participate in the chromatin modulation by snoRNAs (Fig. 3D). Collectively, these findings suggest that snoRNA-chromatin associations do not depend on conventional C/D box snoRNP complex formation or the methyltransferase activity of Fibrillarin.

### Transcription of *SNORD118* increases upon leukemogenesis and decreases during myeloid differentiation

**Fig. 4:**
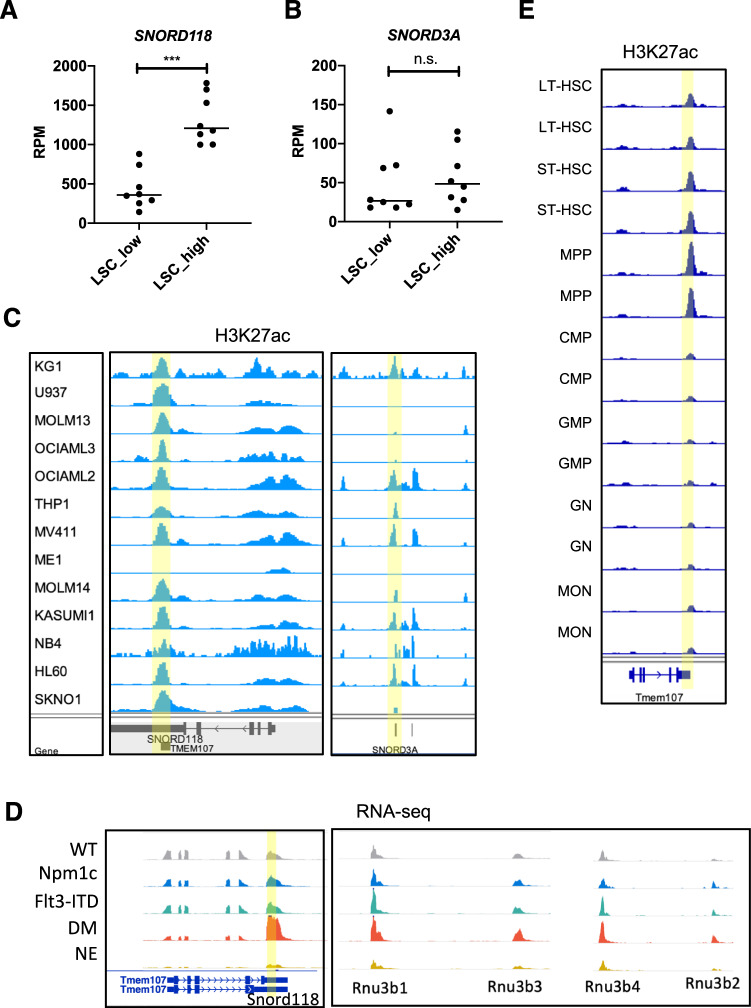
Transcription dynamics of *SNORD118* and *SNORD3A* in normal and malignant hematopoiesis. **A** *SNORD118* or **B** *SNORD3A* expression levels (determined by normalized reads count of small RNA-seq) in AML patients with low (*n* = 8) *versus* high LSC frequency (*n* = 8). **C** Visualization of H3K27ac signals at *SNORD118* locus (left panel) and *SNORD3A* locus (right panel). **D** Visualization of transcription level (determined by RNA-seq) of *SNORD118* (left panel) and *Rnu3b* (right panel) in HSPCs from wildtype (WT), single mutant (*Npm1c* or *Flt3-ITD*), and double mutant (DM) mice, as well as neutrophils (NE) cells. **E** Visualization of H3K27ac signals at *SNORD118* locus across hematopoietic stem cells (HSCs), progenitors, and mature cells. LT-HSC, long-term HSC; ST-HSC, short-term HSC; MPP, multipotent progenitors; CMP, common myeloid progenitors; GMP, granular-macrophage progenitors; GN, granulocytes; MON, monocytes. In **A** and **B**, Student’s unpaired *t*-tests was performed. ***p < 0.001 (two-sided, not multiple testing corrected). Bars represent the mean.

We focused on two C/D-box snoRNAs associated with chromatin, *SNORD118* and *SNORD3A*, and examined their expression dynamics in normal and malignant hematopoiesis. We previously profiled small RNA expression in a cohort of AML patient samples with determined leukemia stem cell (LSC) frequency, using xenotransplantation assays combined with serial dilution analysis [23, 24]. We analyzed the expression levels of *SNORD118* and *SNORD3A* in 16 of these primary samples which were dichotomized into AML with high LSC frequency (*n* = 8) or with low frequency (*n* = 8). *SNORD118* exhibited increased levels in AML specimens with high LSC frequency compared to those with low frequency (Fig. 4A and Supplementary Table 6). In contrast, expression of *SNORD3A* and its homologous genes (including *SNORD3B-1*, *SNORD3B-2*, *SNORD3C* and *SNORD3D*) did not correlate with LSC frequency (Fig. 4B and Supplementary Fig. 4A–D). Further, we detected an active chromatin state marked by H3K27ac at the locus of *SNORD118* (located at 3’UTR of the host gene *TMEM107*) across a panel of human AML cell lines, indicating active transcription of *SNORD118* is common in AML cells (Fig. 4C). Conversely, fewer cell lines exhibited high levels of H3K27ac at the *SNORD3A* locus, suggesting a less pronounced chromatin activation (Fig. 4C). Moreover, in a mouse model that carries the two most prevalent gene mutations in AML, *Npm1c* and *Flt3-ITD* [25], we observed an upregulation of *SNORD118* expression as well as the expression of *Rnu3b* family members (mouse orthologs of human *SNORD3A*) upon leukemia induction by double mutations (DM) (Fig. 4D). Further analysis demonstrated that in wildtype mice the *SNORD118* locus was enriched for H3K27ac in long- and short-term hematopoietic stem cells (HSC), and multipotent progenitors (MPP), but the level of H3K27ac was significantly diminished in committed progenitor and terminally differentiated cells of myeloid lineage (Fig. 4E).

### *SNORD118* and *SNORD3A* in the maintenance of leukemia propagation

**Fig. 5:**
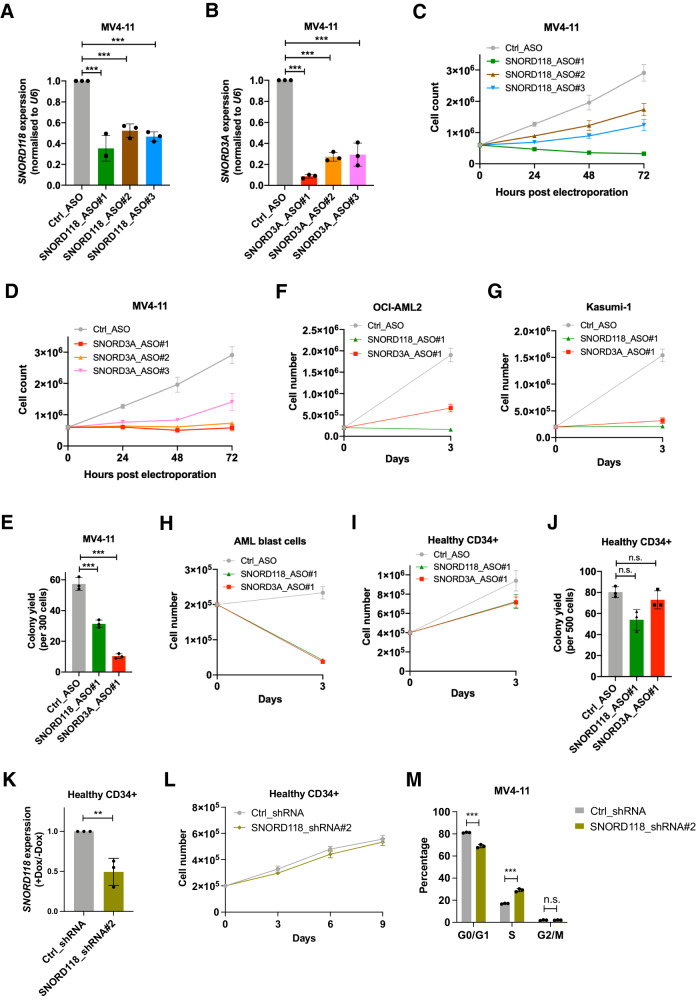
*SNORD118* and *SNORD3A* in the maintenance of leukemia propagation. **A** *SNORD118* or **B** *SNORD3A* expression in MV4-11 cells transfected with control *versus* specific ASOs (n = 3 independent experiments). Expression was relative to U6 and normalized to control ASO. **C** Proliferation assays in MV4-11 cells transfected with control *versus SNORD118* or **D** *SNORD3A* ASOs (*n* = 3 independent experiments). **E** Colony yield of MV4-11 cells transfected with control vesus specific ASOs (*n* = 3 independent experiments). **F** Proliferation assays in OCI-AML2, **G** Kasumi-1, **H** AML patients blast cells or **I** cord blood CD34+ HSPCs transfected with control *versus* specific ASOs. **J** Colony yield of cord blood CD34+ HSPCs control *versus* specific ASOs delivery. **K** *SNORD118* expression in healthy CD34+ peripheral blood cells (PBCs) transduced with control *versus SNORD118* shRNA (oligo #2). Expression was relative to *U6* and normalized to control shRNA. **L** Proliferation assays in healthy CD34+ PBCs transduced with control *versus SNORD118* shRNA (oligo #2). **M** Cell cycle analysis on MV4-11 cells transduced with control versus *SNORD118* shRNA (oligo #2) (*n* = 3 independent experiments). All experiments were performed for three independent replicates, and in **K** and **L** cells were taken from three healthy donors. Student’s unpaired *t*-tests were performed (n.s., not significant; *p < 0.05 ; **p < 0.01; ***p < 0.001; two-sided, not multiple testing corrected). Bars represent the mean and standard deviation.

We analyzed the functional importance of *SNORD118* and *SNORD3A* in leukemia with loss-of-function assays in leukemia cells. Though targeting small ncRNA is technically challenging, antisense oligonucleotides (ASOs) have been demonstrated to be an effective approach [26]. Three ASOs targeting either *SNORD118* or *SNORD3A* demonstrated efficient suppression in MV4-11 cells, resulting in more than 50% reduction of target gene expression compared to ASO control (Fig. 5A, B). Knockdown of *SNORD118* or *SNORD3A* was accompanied by an immediate impairment of cell proliferation over a three-day period (Fig. 5C, D). Besides, cells exhibited weakened colony forming capacity (CFC) upon *SNORD118* or *SNORD3A* depletion (Fig. 5E). The indispensability of *SNORD118* or *SNORD3A* in keeping leukemia cell propagation in liquid culture was recapitulated in two other AML cell lines, the OCI-AML2 and Kasumi-1 cells (Fig. 5F, G). Cross validation using inducible lentiviral shRNA guided knockdown of *SNORD118* also confirmed its essential role in sustaining cell growth and repopulating capacity in MV4-11 cells as well as in two additional AML cell lines, OCI-AML3 and MOLM-13 cells (Supplementary Fig. 5A–I). Moreover, loss of *SNORD118* or *SNORD3A* also impaired proliferation in primary AML bone marrow samples (Fig. 5H). In contrast, depletion of *SNORD118* or *SNORD3A* in cord blood CD34+ hematopoietic stem and progenitor cells (HSPCs) from healthy donors displayed only mild effects on cell growth and colony forming units (Fig. 5I, J). Besides, lentiviral shRNA-mediated knockdown of *SNORD118* also did not significantly impair the proliferation of healthy CD34+ peripheral blood cells for an observation period of 9 consecutive days (Fig. 5K, L).

To uncover how *SNORD118* essentializes leukemia cells propagation, we performed a series of analyses on cell differentiation, cell cycle and apoptosis upon shRNA-mediated *SNORD118* depletion in MV4-11 cells. Myeloid differentiation determined by CD11b and CD64 was not impaired upon *SNORD118* loss (Supplementary Fig. 6A, B). Besides, *SNORD118* knockdown did not alter ATRA-induced neutrophil differentiation in HL-60 leukemia cells (Supplementary Fig. 6C). Interestingly, loss of *SNORD118* resulted in a decrease in G0/G1 phase but an increase in S phase (Fig. 5M), suggesting its anti-proliferative effect is partially mediated by cell cycle arrest. In addition, leukemia cells with *SNORD118* depletion showed a slight increase of apoptosis (Supplementary Fig. 6D). Moreover, *SNORD118* depletion was associated with extensive changes on chromatin accessibility (Supplementary Fig. 6E). These alterations were predominantly enriched at intergenic regions and introns, and were linked to genes including *RARA* and *CDK14* (Supplementary Fig. 6F and Supplementary Table 7). Taken together, these data indicated the importance of *SNORD118* and *SNORD3A* in leukemia maintenance and the potential suitability of *SNORD118* as therapeutic vulnerability since healthy hematopoiesis was less affected upon its loss.

## Discussion

The central dogma of molecular biology describes RNA as an intermediate from DNA to protein; however, regulatory roles of RNA are emerging at multiple levels including chromatin regulation. While microRNAs and lncRNAs are known chromatin regulators, other classes of non-coding RNAs have remained enigmatic in this regard. In this study, we employed a next- generation sequencing (NGS)-based technique called iMARGI to generate an RNA-genome interaction landscape in MV4-11 leukemia cells. As MV4-11 cell line carries both *FLT3* and *MLL* mutations, both were capable of remodeling chromatin landscape, further effort needs to be made to reveal if either mutation can influence RNA-chromatin interactome. Though our RNA-chromatin interactome was profiled in an AML cell line, we anticipate some of our findings may have broader implication beyond AML. For instance, protein-coding mRNAs and lncRNAs were predominantly found at their transcription sites (*cis*-acting), consistent with a recent report of RNA-chromatin interactome profiling in breast cancer cells [27]. However, a few hundred mRNAs exhibited a high frequency of *trans*-interactions. This finding aligns with a recent report that a substantial number of mRNAs associate with *cis*-regulatory elements, indicating their potential for chromatin regulation [27]. There is a notable absence of comprehensive investigations into the collective role of lncRNAs in chromatin regulation. Surprisingly, our study uncovered lncRNAs being less commonly associated with chromatin than mRNAs, as well as less infrequent as *trans*-acting RNA molecules on chromatin. Similar findings were also reported [27], which found that interaction frequencies at transcription site were correlated well with levels of nascent RNA transcripts. Among the *cis*-acting lncRNAs, some were host genes of snoRNAs (e.g. *SNHG* family) and others included *MALAT1*. *MALAT1* has been extensively characterized for its role in chromatin modulation and its localization at nuclear speckles [19, 20, 28]. Although it exhibited a low abundance of *trans*-interactions in the current study, the *MALAT1*-bound sites were found in close proximity to genes encoding signaling factors and *HOXA* genes.

One intriguing finding from our RNA-genome profiling was the notable interaction of snRNA and snoRNA with chromatin in a *trans*-acting manner. These two small ncRNA classes are known for their essential roles in protein synthesis, through guiding pre-mRNA splicing and rRNA processing or modification, respectively [29, 30]. However, recent studies have shed light on novel functions of snRNA and snoRNA in establishing or maintaining chromatin environment, accessibility and structure [27, 31–33]. Our findings align with these discoveries, emphasizing the significance of snRNA and snoRNA in modulating chromatin dynamics in both normal and cancer cells. Although snoRNA-chromatin interaction sites exhibited enrichment for diverse chromatin modifications, questions remain with regard to the establishment of this chromatin association and its impact on chromatin modification processes. Furthermore, our data suggest that the canonical C/D box snoRNP complex may not be directly involved. Future investigations are needed to elucidate the mechanisms underlying snoRNA-mediated chromatin modulation.

Our group has reported an essential role of snoRNAs in ribosome mediated translation and tumorigenesis [24, 34–36]. A recent report uncovered the involvement of two chromatin-associated snoRNAs, *SNORA73A* and *SNORA73B*, in the regulation of differentiation block in leukemia cells [37]. This work highlighted the engagement of chromatin-associated small ncRNAs during cancer development. Consistent with this finding, our study revealed that *SNORD118* and *SNORD3A* expression were dysregulated by leukemogenesis and lineage specification. The functional importance of *SNORD118* and *SNORD3A* in tumorigenesis has also been reported in lung cancer and breast cancer cells [38]. However, their roles in these cancers were primarily associated with pre-rRNA processing and rRNA maturation, which are well-established functions of *SNORD118* and *SNORD3A* in mammalian cells. Moreover, whether these roles are specific to tumors remains to be determined. Of note, our work revealed novel roles of *SNORD118* and *SNORD3A* as chromatin-association factors that are essential for the maintenance of leukemia cell growth. These findings highlight the potential of *SNORD118* and *SNORD3A* as potential targets for leukemia therapy.

## Materials and methods

### Human samples

Human umbilical cord blood cells and bone marrow samples from AML patients as well as healthy donors were obtained at the Heidelberg University Hospital, with prior informed consent in written form in an ethic application (number S-169/2017). The processing and handling of human samples were approved by the Ethics Committee of University Hospital Heidelberg in line with the principles of the Declaration of Helsinki.

### Cell culture

Leukemia cell lines including MV4-11, OCI-AML2, Kasumi-1, OCI-AML3 and MOLM-13 cells were previously purchased from DSMZ. The cells were free from mycoplasma contamination and were recently successfully authenticated for their identity. They were cultured in Gibco RPMI 1640 medium (with L-Glutamine) containing 10% fetal bovine serum (FBS, Gibco) and 1% Penicillin- Streptomycin (P/S, Sigma-Aldrich). Umbilical cord blood, peripheral blood and bone marrow samples were subject to mononuclear cells (MNCs) isolation with Ficoll (GE Healthcare) by density gradient centrifugation. CD34+ cells were isolated from MNCs using MACS system with the CD34 Microbead Kit (Miltenyi Biotec). CD34+ cells and AML blast cells (enriched in MNCs) were cultured in IMDM medium (Gibco), supplemented with 15% FBS, 1% P/S, 100 μmol/L β-mercaptoethanol (Thermo Fisher), 500 nmol/L StemRegenin 1 (STEMCELL Technologies), 1 μmol/L UM729 (STEMCELL Technologies), and a cocktail of cytokine mix (all from PeproTech) containing 100 ng/mL SCF, 20 ng/mL IL3, 50 ng/mL FLT3L, 20 ng/mL G-CSF.

### in situ mapping of RNA–genome interactome (iMARGI)

iMARGI was carried out following an established protocol [17, 18]. In brief, ten million of MV4-11 cells were harvested from culture and were crosslinked using 1% formaldehyde (Thermo Fisher Scientific) at room temperature for 10 min and quenched with glycine (Sigma-Aldrich). Cells were lysed for nuclei isolation, followed by RNA and DNA fragmentation in the nuclei using RNase I and restriction enzyme AluI. Subsequently, a RNA/DNA hybrid linker was ligated to the fragmented RNA and then to the DNA with spatial proximity. This was followed by crosslinking reversal, nucleic acids isolation, and unligated linkers removal. Magnetic streptavidin beads were utilized to further pull down the RNA-linker-DNA ligation fragments. The RNA elements were reverse transcribed into cDNA, and the new double stranded fragments were heat denatured to release the single strand containing DNA-linker-cDNA. This single stranded DNA-linker-cDNA was further subject to circularization, oligo annealing to form a BamHI site, re-linearisation by BamHI digestion. As the linearized DNA contained 5’ and 3’ sequences compatible with Illumina sequencing, samples were amplified with PCR and size-selected for pair-end sequencing (2 x 75 bp) on Illumina NextSeq at the EMBL Genecore Genomics Facility. In total four libraries were constructed and sequenced, generating 170-450 million paired reads per library. For calling all valid RNA-chromatin interactions, reads from each library were processed with iMARGI-Docker following developer’s instructions as described [17]. Data were aligned to the hg38 reference genome. The valid RNA-chromatin interactions were merged for downstream analysis.

### qPCR analysis on iMARGI library

qPCR assays were performed to verify the trans-interactions between candidate RNAs and genomic loci. Primer sets were designed based on the iMARGI data and available in Supplementary Table 8. In principle, the forward or reverse primers were designed to target the candidate RNA or genomic loci, respectively, using Primer 3 (https://primer3.ut.ee/). Two primer sets targeting on non-interacting regions were also included in the analysis. Meanwhile, primer sets to quantify the total RNA fragments captured by iMARGI were also designed. Enrichment of RNA-chromatin interaction frequency was calculated by the ratio of PCR amplification of RNA-DNA product to PCR amplification of candidate RNA.

### Chromatin Isolation by RNA purification (ChIRP)

ChIRP was performed as previously described [22] with modifications. Briefly, 20~30 million MV4-11 cells were collected per IP condition. Crosslinking was executed with 1% glutaraldehyde (Sigma-Aldrich) for 10 min at room temperature and quenched by addition of glycine (Thermo Fisher Scientific). Cells were then lysed and lysate was sonicated using a Bioruptor Standard (Diagenode), with a setting of 30 s ‘ON’ and 45 s ‘OFF’ pulse intervals for 1~1.5 h in a 4 °C water bath, till lysate turned clear. Next, biotinylated DNA probes (of control, *SNORD3A*, or *SNORD118*, synthesized by Integrated DNA Technologies) were added to hybridize with individual target RNA. RNA-associated chromatin was subsequently captured using magnetic dynabeads and underwent thorough washes. Afterwards, the enriched chromatin was divided into two fractions for separate crosslinking reversal and isolation of RNA and DNA with Trizol (Invitrogen) and Phenol:Chloroform:Isoamyl Alcohol (25:24:1, v/v) (Sigma-Aldrich), respectively. The extracted RNA was reverse transcribed and was examined for capture efficiency and specificity by running Real-Time PCR on control and target gene sites. The DNA elute was subject to NGS library preparation using NEBNext Ultra II DNA Library Prep Kit for Illumina (New England Biolabs). The library was individually barcoded and pooled to load onto Illumina NextSeq for single-read sequencing (1 × 50 bp) at the EMBL Genecore Genomics Facility.

### Chromatin immunoprecipitation coupled with sequencing (ChIP-seq)

ChIP was performed with one million or five million cells per IP condition, using an iDeal ChIP- seq kit for Histones (for H3K27ac, H3K9me3) or for Transcription Factors (for FBL) (Diagenode) following the manufacturer’s recommendations. Briefly, cells were crosslinked with formaldehyde (Thermo Fisher Scientific) at a final concentration of 1% for 10 min and then quenched by addition of Glycine (Thermo Fisher Scientific). Cells were then lysed to harvest chromatin-containing nuclei. Chromatin was sheared using the Bioruptor Standard (Diagenode) for 20 cycles (each cycle for 30 s ‘ON’ and 45 s ‘OFF’) in a 4 °C water bath. Subsequently, immunoprecipitation, washing, decrosslinking and DNA elution were performed as per the manufacturer’s protocol, using 1 µg anti-H3K27ac antibody (Abcam, #ab4729), 1 µg anti-H3K9me3 antibody (Diagenode, #c15410003), or 5 µg anti-FBL antibody (Abcam, #ab5821). Besides, 10% of the sheared chromatins were kept aside as input samples to perform decrosslinking and DNA isolation. Afterwards, ChIP DNA or input DNA was processed for library preparation using NEBNext Ultra II DNA Library Prep Kit for Illumina (New England Biolabs) following standard procedures from the manufacturer. Libraries were pooled for single-read (1 × 50 bp) sequencing on an Illumina NextSeq at the EMBL Genecore Genomics Facility.

### ATAC-seq

ATAC-seq was carried out as previously described [25]. In brief, 100,000 freshly isolated cells were pelleted and washed with ice-cold PBS. The cell pellet was collected and was resuspended in Lysis buffer. The crude nuclei pellet was preserved for tagmentation using ATAC-seq kit (Active Motif) for 30 min incubation at 37 °C. Afterwards, transposed DNA was purified using with MinElute PCR Purification kit (Qiagen). Eluted DNA was amplified by setting the PCR cycling as below: step 1. 72 °C 5 min, 98 °C 30 s; step 2. 10 cycles: 98 °C 10 s, 63 °C 30 s, and 72 °C 1 min; step 3. 72 °C 5 min. PCR products were again purified with Qiagen MinElute PCR Purification kit. The quantity and quality of library DNA was measured using Qubit and and Bioanalyzer 2100 system with a High Sensitivity DNA chip (Agilent Technologies). The pooled libraries were sequenced for paired-read (2 × 75 bp) sequencing on an Illumina NextSeq at the EMBL Genecore Genomics Facility. Two biological replicates were performed independently for each treatment.

### ChIRP, ChIP-seq and ATAC-seq analysis

The sequencing data from ChIRP, ChIP-seq and ATAC-seq assays were analyzed in a similar fashion as previously described [25, 39]. The sequencing reads were first aligned to the human reference genome (hg38) using Bowtie and subject to QC check. Repeat reads were removed using PICARD tools. To visualise the peak profiles accross the genome, bigwig files were generated from uniquely mapped reads of each sample and were normalized to library size (as counts per million, CPM) by running bamCoverage from deepTools. Peaks were called using MACS2 with a *P* value cutoff of 1 × 10^-9 and with setting ‘nomodel’. The enrichment of chromatin modifications (H3K27ac or H3K9me3) or target RNA binding at specific genomic regions was evaluated using deepTools packages. The reads signals were calculated into a matrix of enrichment scores using computeMatrix, and such a matrix was further illustrated by either a heatmap or a plot of average profile using plotHeatmap or plotProfile, respectively. ATAC-seq differential analysis was performed using using edgeR, with significant changes being defined by FDR value < 0.05 and log2FC ≥ 1 (gain or loss) between control and SNROD118 shRNA expressing cells. Genomic annotation of differential peaks was analyzed with the help of a web tool – PAVIS (https://manticore.niehs.nih.gov/pavis2/).

### Electroporation with antisense oligonucleotides (ASOs)

The sequences of ASOs were listed in the Supplementary Table 8. ASOs were electroporated into cells with the Neon transfection system (Invitrogen) following the manufacturer’s instruction. Briefly, 2.5 ×10^5 cells were washed with PBS (without Ca^2+^ and Mg^2+^), pelleted down and resuspended in 11 µL resuspension buffer R (for leukemia cell lines) or buffer T (for primary cells). One μL of 500 µM ASOs were added into the cells, mixed well and incubated for 10–15 min with at room temperature. The cell-ASOs mixture was used for electroporation on the Neon system with a 10-µL Neon Tip, with a setting of pulse voltage at 1400 V, Pulse width at 25 ms, and number of pulses at 1. Afterward, cells were transferred into 500 μL medium (RPMI 1640 with 10% FBS, without antibiotics) on a 24-well plate for culturing under a normal condition (with 5% CO_2_, good humidity at 37 °C).

### Cell proliferation

The electroporated cells were evaluated for ex vivo proliferation at a 24-h interval for three consecutive days post electroporation. Viable cells were counted with trypan blue on a hemocytometer. For shRNA transduced cells, 5 × 10^5 cells were seeded in 1 mL medium and doxycycline was added at a final concentration of 100 ng/mL to induce shRNA expression. Cell number was examined every 3 days till day 12 post doxycycline supplement. Three biological replicates were performed independently.

### Flow cytometry analysis on cell cycle, apoptosis and myeloid differentiation

Cell cycle and apoptosis measurement was performed as previously described [40]. In brief, for cell cycle analysis, at first cells were fixed with 2.5 volumes of ethanol and incubated on ice for 15 min, followed by treatment with 50 µg/mL propidium iodide (PI; BD Biosciences) for staining along with 0.1 mg/mL RNase A and 0.05% Triton X-100 (Sigma-Aldrich), incubation at 37°C for 40 min. The samples were then resuspended in PBS and were measured on flow cytometer. Apoptosis assays was carried out with Annexin V-FITC and PI staining according to the manufacturer’s protocol (BD Biosciences), and were measured on flow cytometer. Myeloid differentiation was evaluated by staining cells with PE-CD11b (BioLegend, #101208) and FITC-CD64 (BioLegend, #399506) antibodies and measured on flow cytometer. All the data analysis was performed with FlowJo 10.8.1 software.

### Colony forming unit (CFU) assays

At 4 h post electroporation, 200 cells were harvested and plated per dish in duplicate on methylcellulose medium. The same number of shRNA transduced cells were taken for CFU assays on methylcellulose medium containing 100 ng/mL doxycycline. Colonies were scored manually using microscopy after 8–10 days. MethoCult H4230 and MethoCult GF H4434 (both from STEMCELL Technologies) were used for leukemia cell lines and primary cells, respectively. Three independent biological replicates were performed.

### Lentiviral shRNA mediated gene knockdown

The inducible lentiviral shRNA plasmids were generated by cloning of target shRNA oligos into Tet-pLKO-puro vector (cat. 21915, Addgene) following the provider’s instruction. Lentiviral particles were produced by co-transfection of shRNA plasmids with psPAX and pMDG.2 in 293 T cells using Turbofect Transfection Reagent (Thermo Scientific). Cells were infected by shRNA lentivirus via centrifugation for 2500 rpm, 60 min at 32 °C in the presence of 8 μg/mL polybrene (cat. TR-1003-G, Millipore). At 72 h post transduction, cells were selected with 2 μg/mL puromycin (Sigma-Aldrich) for 72 h. The knockdown effect was evaluated by treating the cells with 100 ng/mL doxycycline for three days, and then isolating RNAs for reverse transcription with SuperScript IV Synthesis Kit (Thermo Scientific) and quantitative Real-Time PCR (qRT-PCR) analysis. The sequences of shRNA oligos as well as qRT- PCR primers were listed in Supplementary Table 8.

### Statistics and reproducibility

Statistical analyses in the studies were specified in details in figure legends. Sample sizes were chosen on power calculations of expected differences. Randomization was applied to gene loss-of- function experiments. The investigators who performed experiments were not blinded to allocation during experiments and outcome assessment. No data were excluded for analysis. Our data subjected for Student’s unpaired *t*-test were estimated for a normal distribution and have been performed as two-tailed comparison with GraphPad Prism (v.8.2.1). Number of independent experiments or independent samples were specified in figure legends. Representative data or images were replicated in at least three independent experiments.

### Supplementary information

Supplementary Figures and Tables legend

Supplementary Figures

Supplementary Table 1

Supplementary Table 2

Supplementary Table 3

Supplementary Table 4

Supplementary Table 5

Supplementary Table 6

Supplementary Table 7

Supplementary Table 8

## Data Availability

All sequencing raw data and processed data have been deposited in the GEO database under the series: GSE267083 for iMARGI, GSE267081 for ChIRP, GSE267136 for ChIP-seq, and GSE267080 for ATAC-seq, with no restrictions to access. All supporting data derived from the sequencing analysis to assist understanding of the results and discussions in the paper were provided in several supplementary tables. The studies have also re-analyzed multiple datasets that are publicly available: the ChIP-seq on H3K4me3 (GSM2108040), H3K36me3 (GSM1897375), H3K79me2 (GSM2108041), H3K9ac (GSM2136947), H3K27ac (GSM2108039), H3K27me3 (GSM1513828), BRD4 (GSM2716694), and RNA Pol II (GSM2716705), all in MV4-11 leukemia cells.
