# Supplementary material for: The landscape of RNA-chromatin interaction reveals small non-coding RNAs as essential mediators of leukemia maintenance
Source: Leukemia. 2024 Jun 28;38(8):1688–98. doi: 10.1038/s41375-024-02322-7 (PMC11286530; doi:10.1038/s41375-024-02322-7)
Supplement: Supplementary file 1 — Supplementary Figures and Tables legend [file 41375_2024_2322_MOESM1_ESM.docx]

## The landscape of RNA-chromatin interaction reveals small non-coding RNAs as essential mediators of leukemia maintenance

Haiyang Yun^1,2,3*^, Julian Zoller^1^, Fengbiao Zhou^1,3^, Christian Rohde^1,3^, Yi Liu^1,3^, Maximilian Felix Blank^1,3,4^, Stefanie Göllner^1^, Carsten Müller-Tidow^1,3,5^*

^1^Department of Medicine V, Hematology, Oncology and Rheumatology, University Hospital Heidelberg, Heidelberg, Germany

^2^The Robert Bosch Center for Tumor Diseases, Stuttgart, Germany

^3^Molecular Medicine Partnership Unit, European Molecule Biology Laboratory (EMBL), Heidelberg, Germany

^4^Division Proteomics of Stem Cells and Cancer, German Cancer Research Center (DKFZ), Heidelberg, Germany

^5^National Center for Tumor Diseases (NCT), Heidelberg, Germany

*Corresponding authors

**Correspondence:**

Haiyang Yun, PhD, and Carsten Müller-Tidow, MD, Department of Internal Medicine V, Heidelberg University Hospital, 69120 Heidelberg, Germany. Phone: 0049-6221-568000; E-mail: Haiyang.Yun@bosch-health-campus.com; [Carsten.Mueller-Tidow@med.uni-heidelberg.de](mailto:Carsten.Mueller-Tidow@med.uni-heidelberg.de)

**Competing interests**

The authors declare no conflict of interest.

## Supplementary Figure legend

## Supplementary Fig. 1: Identification of chromatin-associated protein-coding mRNAs or lncRNAs in MV4-11 cells.

## A Distribution of chromatin-associated protein-coding mRNAs or (B) lncRNAs with high to low percentage of *trans*-interactions. *Trans*-acting RNAs are marked in red, defined by a cut-off at 20% for trans-interactions. C Gene ontology analysis of *trans*-acting protein-coding mRNAs. D Enrichment of chromatin interaction frequencies by *CEBPA*, (E) *JUN* or (F) *MALAT1* mRNA by qPCR analysis. G Circos plot showing genome-wide interactions between *CEBPA*, (H) *JUN* or (I) *MALAT1* mRNA and chromatin in HEK293T cells. Red arcs, *trans*-interactions; green arcs, *cis*-interactions; blue track, interaction frequencies.

## Supplementary Fig. 2: Identification of chromatin-associated snoRNA and snRNAs.

## A Distribution of chromatin-associated snoRNAs or (B) snRNAs with high to low percentage of trans- interactions. *Trans*-acting RNAs are marked in red, defined by a cut-off at 20% for trans-interactions. C Exemplar loci showing chromatin occupancy by *U1* and (D) *SNORD3A*. Left, proximal region; Right, distal region.

## Supplementary Fig. 3: Enrichment of chromatin states at snRNA or snoRAN-chromatin interaction sites.

## A Enrichment of various histone modifications and occupancy of RNA Pol II and BRD4 at snRNA- or (B) snoRNA-chromatin interaction sites.

## Supplementary Fig. 4: Expression of *SNORD3A* homologous genes in AML patients with low or high LSC frequency.

## A *SNORD3B-1*, (B) *SNORD3B-2*, (C) *SNORD3C* or (D) *SNORD3D* expression levels (determined by normalized reads count of small RNA-seq) in AML patients with low (n = 8) *versus* high LSC frequency (n = 8). Student’s unpaired t-tests were performed (n.s., not significant). Bars represent the mean.

## Supplementary Fig. 5: Leukemia cell propagation upon shRNA-mediated loss of *SNORD118*.

## A *SNORD118* expression, (B) Proliferation assays or (C) Colony forming units assays in MV4-11 cells transduced by control *versus* two *SNROD118* shRNAs. D *SNORD118* expression, (E) Proliferation assays or (F) Colony forming units assays in OCI-AML3 cells transduced by control *versus* two *SNROD118* shRNAs. G *SNORD118* expression, (H) Proliferation assays or (I) Colony forming units assays in MOLM-13 cells transduced by control *versus* two *SNROD118* shRNAs. In (A), (D) and (G), expression was relative to *U6* and normalized to control shRNA. All experiments were performed for three individual replicates. Statistical analyses were performed by running Student’s unpaired t-tests (n.s., not significant; *, p < 0.05 ; **, p < 0.01; ***, p < 0.001; two-sided, not multiple testing corrected). Bars represent the mean and standard deviation.

## Supplementary Fig. 6: Molecular alterations induced by shRNA-mediated loss of *SNORD118*.

## A Representative plot or (B) quantification of myeloid differentiation markers expression (FITC-CD64 and PE-CD11b, measured with flow cytometry) in MV4-11 cells transduced with control *versus SNORD118* shRNA (oligo #2). C Representative plot of CD11b expression level in HL-60 cells transduced with control *versus SNORD118* shRNA (oligo #2) and treated with 1μM ATRA for 5 days. D Apoptosis measurement in MV4-11 cells transduced with control *versus SNORD118* shRNA (oligo #2). E MA plot showing differential ATAC-seq peaks in MV4-11 cells transduced with control *versus SNORD118* shRNA (oligo #2). Differential threshold was set at FDR < 0.05 and log2FC >=1. Red, increase in *SNORD118* shRNA; Blue, decrease in *SNORD118* shRNA. All experiments were performed for three individual replicates. F Genomic annotation features of differential ATAC-seq peaks from (E). Up- and down-regulated peaks were annotated separately. Upstream was defined within 5kb from transcription starting site, whereas downstream was 1kb from gene end.

## Supplementary Tables

**Supplementary Table 1: Chromatin interactions associated with snRNAs and snoRNAs.**

**Supplementary Table 2: Trans-acting snRNAs and snoRNAs.**

**Supplementary Table 3: Chromatin regions associated with *U1*, *SNORD3A*, and *SNORD118* revealed by ChIRP.**

**Supplementary Table 4: Active chromatin regions (C1) of snoRNA-chromatin interaction sites.**

**Supplementary Table 5: Fibrillarin-bound sites across genome in MV4-11 (shCtrl) cells. Supplementary Table 6: Normalized RNA-seq reads (in RPM) of *SNORD118* and *SNORD3A* in AML patients with low or high LSC frequency.**

**Supplementary Table 7: Genes annotated to the differential ATAC-seq peaks in MV4-11 cells expressing control or *SNORD118* shRNA.**

**Supplementary Table 8: Sequences of oligonucleotide used in this study.**
