# Supplementary material for: The landscape of RNA-chromatin interaction reveals small non-coding RNAs as essential mediators of leukemia maintenance
Source: Leukemia. 2024 Jun 28;38(8):1688–98. doi: 10.1038/s41375-024-02322-7 (PMC11286530; doi:10.1038/s41375-024-02322-7)

Supplementary Figure 1

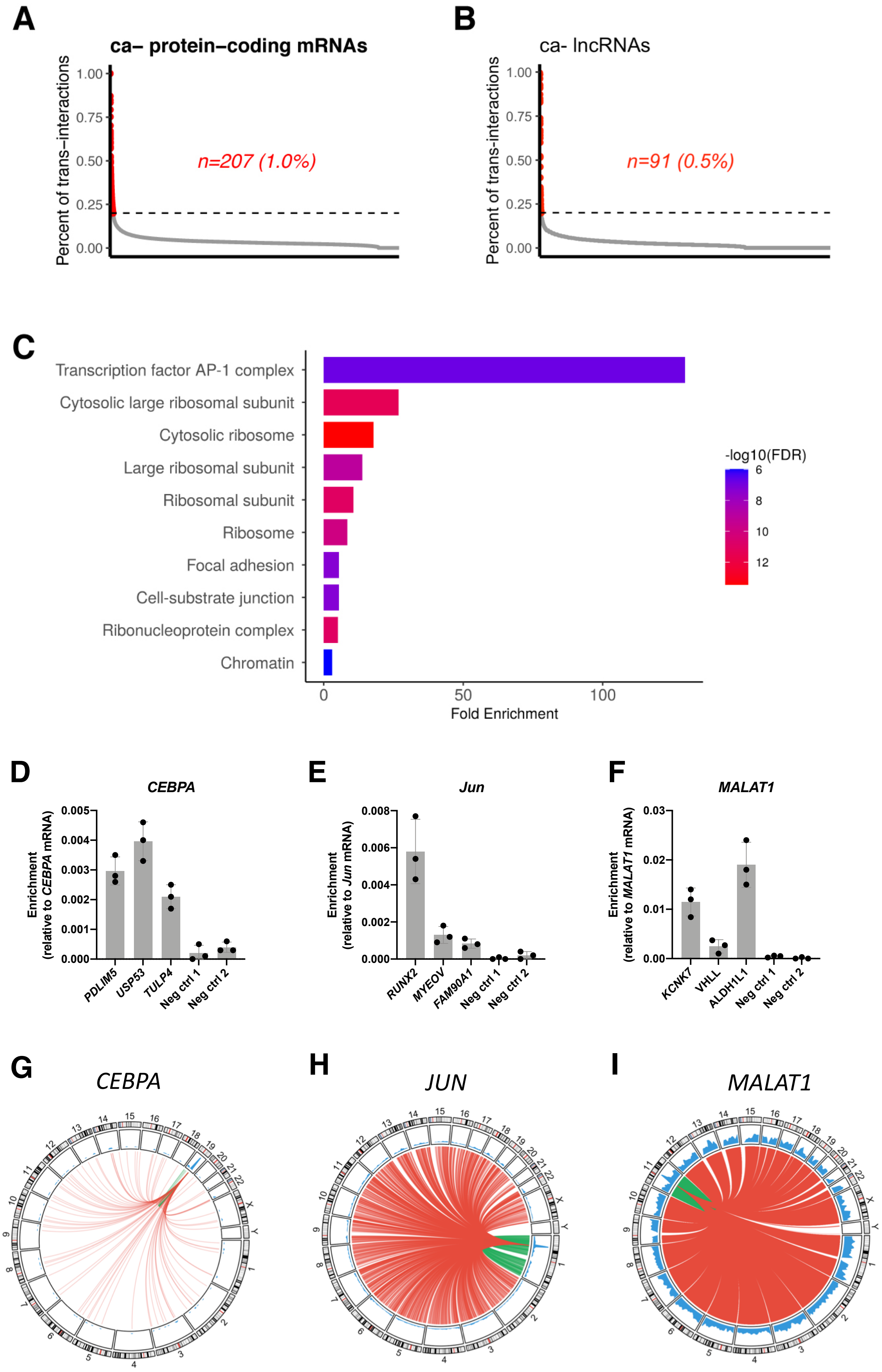

Supplementary Figure 2

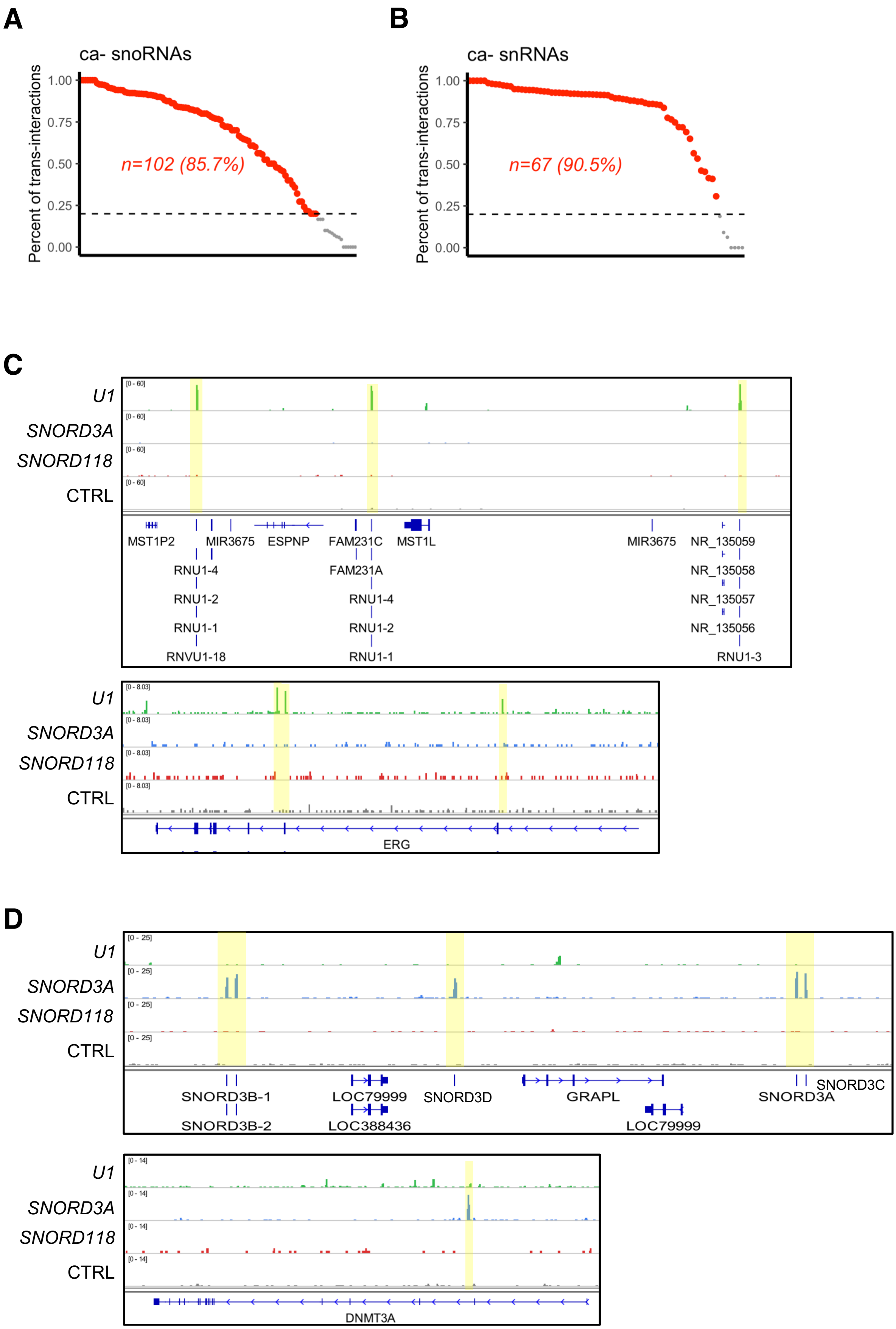

Supplementary Figure 3

A

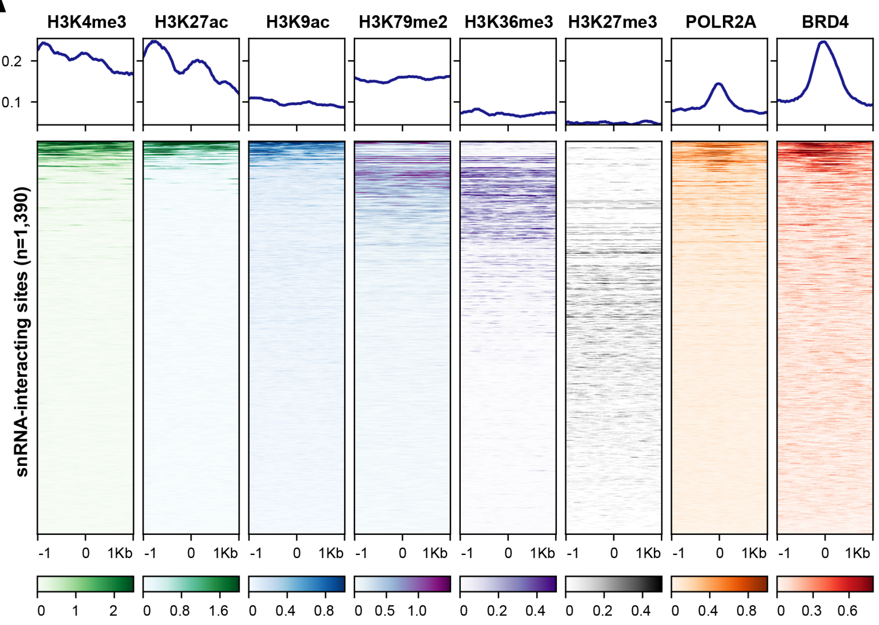

B

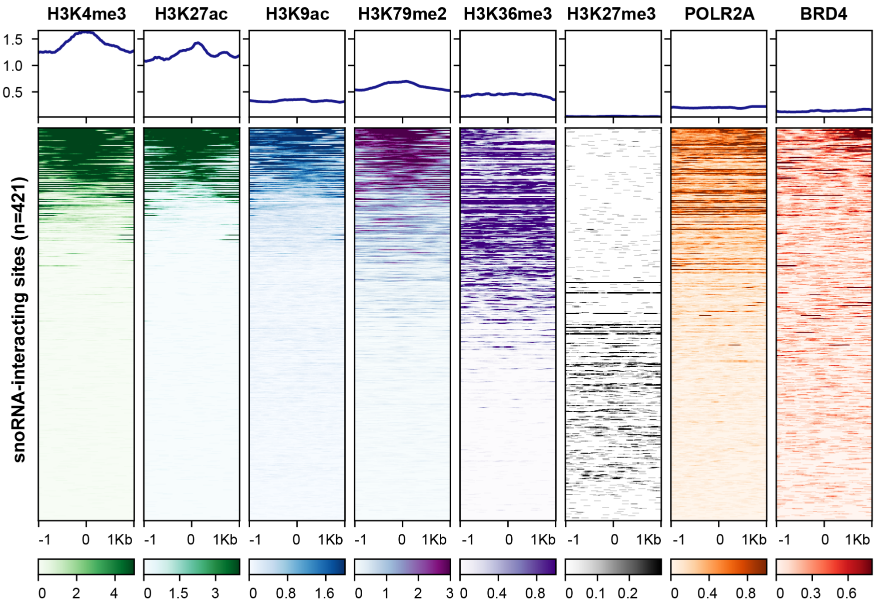

C

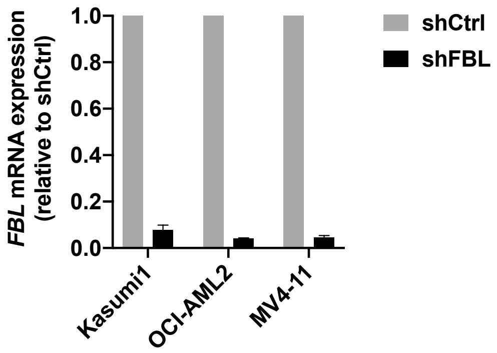

Supplementary Figure 4

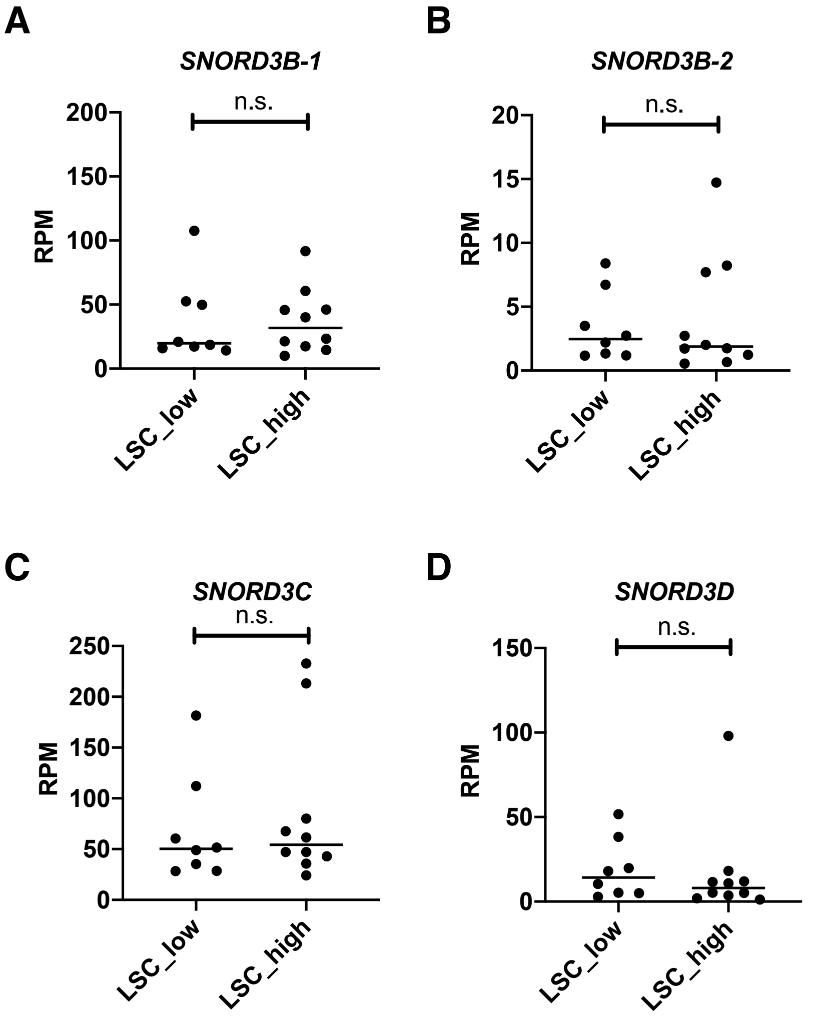

Supplementary Figure 5

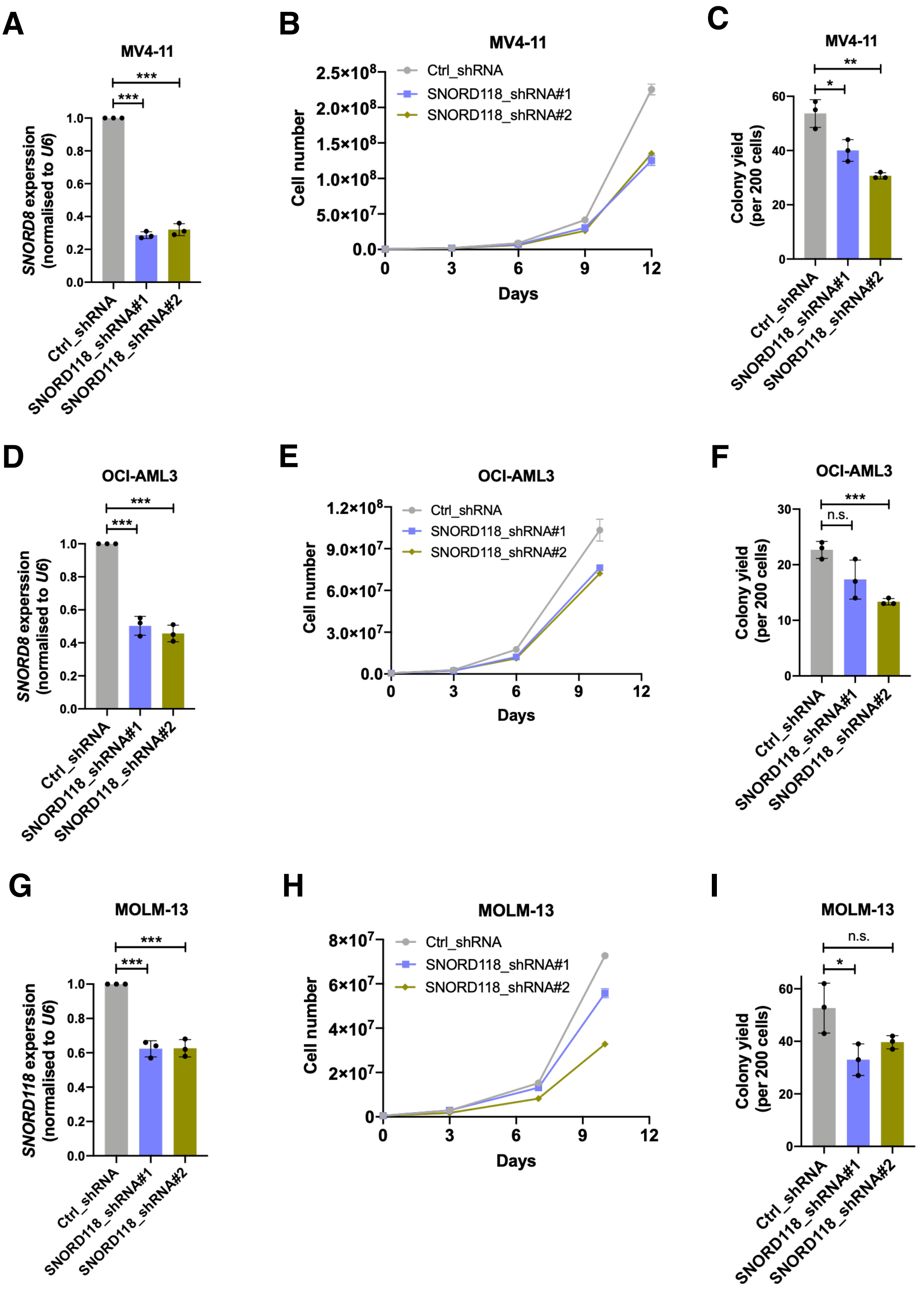

Supplementary Figure 6

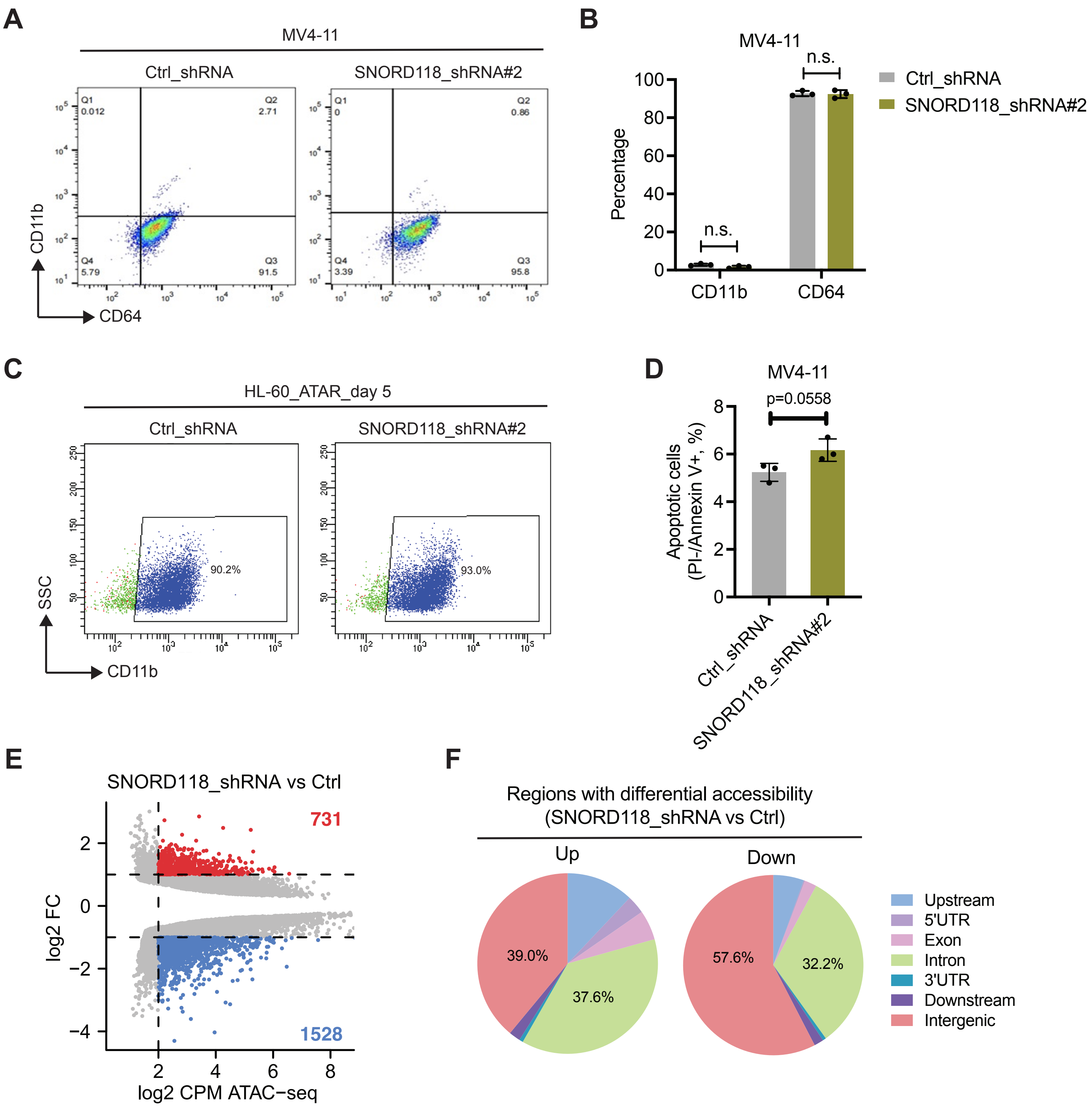

Supplement: Supplementary file 2 — Supplementary Figures [file 41375_2024_2322_MOESM2_ESM.pdf]
